# SUPPORTING DECISIONS ABOUT END-OF-LIFE CARE IN DEMENTIA: FEASIBILITY OF A DECISION AID

**DOI:** 10.1093/geroni/igac059.942

**Published:** 2022-12-20

**Authors:** Nathan Davies, Narin Aker, Victoria Vickerstaff, Elizabeth Sampson, Greta Rait

**Affiliations:** UCL, London, England, United Kingdom; UCL, London, England, United Kingdom; UCL, London, England, United Kingdom; UCL, London, England, United Kingdom; UCL, London, England, United Kingdom

## Abstract

Decisions about end-of-life care are often left to family caregivers to make with professionals. Caregivers find these decisions difficult. A decision aid is one option to support family caregivers. We aimed to test the acceptability and feasibility of a co-produced decision aid for family carers of people with severe dementia or those towards the end of life in the UK. We aimed to recruit 30 family caregivers for a 6 month pre-post test feasibility study. Primary outcome was the feasibility of the study. We included quantitative measures at baseline, 3 month and 6 months, including: Decisional Conflict scale (DCS) and Kessler Psychological Distress Scale (K10). We recruited 28 carers (93% target), 26 completed baseline and 20 completed 6 month follow up. All outcomes changed indicating improvement at 6 months. Qualitative interviews reported the decision aid was acceptable. We met our success criteria, the study is feasible and the decision aid acceptable to caregivers.

